# Factors influencing fecundity in experimental crosses of water lotus (*Nelumbo nucifera* Gaertn.) cultivars

**DOI:** 10.1186/1471-2229-12-82

**Published:** 2012-06-07

**Authors:** Nian-Jun Teng, Yan-Li Wang, Chun-Qing Sun, Wei-Min Fang, Fa-Di Chen

**Affiliations:** 1 College of Horticulture, Nanjing Agricultural University, Nanjing, 210095, China; 2 Zhenjiang Agricultural Research Institute, Jurong, Jiangsu, 212400, China

## Abstract

**Background:**

Breeding programs for the water lotus (*Nelumbo nucifera*) are hampered by an inability to account for variation in seed set associated with crosses between different cultivars. We studied seed set in two reciprocal crosses between lotus cultivars (‘Guili’ × ‘Aijiangnan’ and ‘Molingqiuse’ × ‘Qinhuaiyanzhi') to obtain insights into factors that govern fecundity in these experimental hybrids. Pollen viability, stigma receptivity and embryo development were compared for each hybrid and reciprocal cross.

**Results:**

Pollen viability of the individual cultivars ranged from 4.1% to 20.2%, with the highest level (>11.9%) for all cultivars observed from the earliest collected grains (05:00–06:00 a.m.). Stigmatic pollen germination peaked at 4 h after pollination and varied from 4.8 to 60.6 grains per stigma among the crosses. Production of normal embryos ranged from 7.6% to 58.8% at 1 d after pollination and from 0 to 25% by 11 d after pollination. Seed set in crosses (0.2–23.3%) was generally lower than in open-pollinated plants (8.4–26.5%). Similar to the germination results, seed set was substantially reduced in both reciprocal crosses.

**Conclusions:**

These results suggested that poor pollen fertility, low stigma receptivity, and embryo abortion were responsible for the failure of the crosses ‘Molingqiuse’ × ‘Qinhuaiyanzhi’, ‘Qinhuaiyanzhi’ × ‘Molingqiuse’, and ‘Aijiangnan’ × ‘Guili’.

## Background

The Nelumbonaceae is a family of perennial, aquatic, herbaceous angiosperms, consisting of the two species *Nelumbo nucifera Gaertn*. and *N. lutea* (Willd.) Pers. *Nelumbo nucifera*, also called the Indian or sacred lotus, is found throughout Asia and Australia, whereas *N. lutea*, also known as the American lotus or water chinquapin, occurs in eastern and southern North America [1,2]. Fossilized leaves of *N. nucifera* show that wild populations were distributed previously in eastern Asia including China, India and Japan about 50 million years ago. Wild populations of *N. nucifera* in China are now mainly distributed in the provinces of Helongjiang, Jilin, and Sandong. *Nelumbo nucifera* was cultivated in China more than 2000 years ago. The current main cultivation areas in China comprise the provinces of Jiangsu, Zhejiang, Hubei, Anhui, Guangdong, Hunan, Fujian, and Jiangxi. This species is propagated vegetatively by rhizomes in commercial cultivation, and by seeds for breeding purposes [3]. As an economically important aquatic plant in China, *N. nucifera* cultivars are classified into three groups: rhizome lotus, seed lotus and water lotus or ornamental lotus according to their use and morphological features [4,5]. Water lotus is among the 10 most popular traditional flowers in China, and is an important ornamental plant, widely cultivated in gardens prized for the beauty of its flowers as well as its ability to purify pond water [6,7]. With the recent rapid growth in the Chinese economy and increased standard of living, the demand for new lotus cultivars with novel characteristics has also increased. Therefore, Chinese breeders are making great efforts to develop new cultivars using methods such as artificial interspecific hybridization, mutation techniques, and multiploid approaches [7-9]. Hybridization is the most widely used breeding technique and the most efficient method by which to produce new water lotus cultivars. However, the existence of incongruity barriers often makes many water lotus crosses difficult, and thus raising hybrids may be hard to achieve, thereby seriously reducing the breeding efficiency [7]. Few previous studies have been carried out to investigate this problem, and thus the factors that influence the breeding efficiency of water lotus remain unclear.

In general, the characteristics of parental reproductive systems and their interactions are closely related to the breeding efficiency of plant hybridization. Consequently, parental reproductive systems and post-pollination phenomena have been examined in many crops, including *Chrysanthemum grandiflorumLeymus chinensisPhaseolus vulgaris*, and *Fragaria ananassa*[10-13]. Most of these studies have successfully unraveled the factors that influence seed production and breeding efficiency. Following on from these previous studies, we set out to investigate systematically the reproductive processes following artificial pollination in two reciprocal crosses between water lotus cultivars, including pollen viability of the male parents, germination behavior of pollen grains on the stigma, and embryo development. The aim of the present study was to reveal the main causes that lead to low seed set in water lotus crosses, to overcome reproductive barriers and improve the efficiency of water lotus cross-breeding in the near future.

The cultivars used in the present study were developed about 10 years ago through cross-breeding. Anthesis of lotus flowers is very specific and complex. The pistil of *N. nucifera* flowers is shaped like a flat-topped cone and contains 20–35 ovules when fully developed. Surrounding the pistil are 200–300 yellow stamens, and both the male and female reproductive organs are protected by 10–35 petals and sepals. Anthesis of individual flowers usually lasts about three days on sunny days. On the first day, the flower begins to open at about 03:00 in the morning and attains its maximum diameter (about 6 cm) at about 05:30. The flower remains at this diameter until about 09:00, after which the flower gradually closes and is completely closed at about 15:00. Substantial fluid is secreted on the stigma surface from 04:00 to 06:00 and the stigma is a bright yellow color, which indicates that the stigma receptivity is high during this period. The anthers do not dehisce on the first day, therefore the pistils mature before the stamens in an individual flower (termed ‘protogyny’). On the second day of anthesis, the process of flower opening is very similar to that on the first day and a maximum diameter of about 10 cm is attained. The anthers dehisce to release quantities of mature pollen from about 04:00 to 05:30. The stigma surface becomes dry and brown, which indicates the loss of stigma receptivity. On the third day of anthesis, the flower begins to open at about 05:00 and is fully open with a maximum diameter of more than 12 cm in diameter at about 10:00. Subsequently, the petals and stamens abscise. The stigma becomes severely dry and brown, and its receptivity is completely lost. An individual flower usually produces about one million pollen grains. Thus the pollen:ovule ratio (P/O) is 30,000-50,000.

The study site is about 33 hectares in area and contains many pools. Many lotus cultivars, including the four included in the present study, were planted in the pools. Therefore, all of the lotus cultivars growing in at study site were possible sources of pollen for “naturally pollinated flowers” in the present study. Bees, flies and beetles may be effective pollinators at the study location.

## Methods

### Experimental materials

Two reciprocal crosses (i.e., four crosses in total) were performed using four cultivars of water lotus, i.e. *N*. *nucifera* ‘Guili’ (pollen receptor) ×  *N*. *nucifera* ‘Aijiangnan’ (pollen donor) (hereafter referred to as GA), *N*. *nucifera* ‘Aijiangnan’ (pollen receptor) ×  *N*. *nucifera* ‘Guili’ (pollen donor) (AG), *N. nucifera* ‘Molingqiuse’ (pollen receptor) ×  *N. nucifera* ‘Qinhuaiyanzhi’ (pollen donor) (MQ), and *N. nucifera* ‘Qinhuaiyanzhi’ (pollen receptor) ×  *N. nucifera* ‘Molingqiuse’ (pollen donor) (QM). About 100 plants of each cultivar were grown in pools in Nanjing Yileen, Zhujiang town, Pukou district, Nanjing, China (32°07' N, 118°62' E). The four cultivars are diploid and have a chromosome number of 16 (2 *n* = 2 *x* = 16). These specific cultivars were selected for two main reasons. First, the ornamental values of ‘Aijiangnan’ and ‘Guili’ are complementary, for example the flower color of ‘Aijiangnan’ is butter-yellow and that of ‘Guili’ is pink. If these two cultivars can be crossed successfully, it should be possible to obtain genotypes with novel traits. This is also the case for ‘Molingqiuse’ and ‘Qinhuaiyanzhi’. Second, our preliminary studies indicated that seed set differed among these crosses, which motivated us to examine the factors that influence the fecundity of these crosses. In the present study, flowers from multiple plants of each cultivar were used, because one plant was unable to produce a sufficient number of flowers for the present experiment. However, within each cultivar the plants had an identical genetic background, because they were propagated vegetatively from rhizomes. In addition, before we formally carried out the present study, we performed preliminary tests to examine if there was a significant difference in behavior between flowers from the same plant and between flowers those from different plants within a given cultivar. No significant differences were observed.

### Determination of pollen viability

Previous studies have shown that lotus pollen is only viable for a few hours after anther dehiscence and that it is difficult to determine lotus pollen viability [3]. In the present study, we systematically estimated lotus pollen viability using a variety of methods, such as fluorescein diacetate (FDA), triphenyltetrazolium chloride (TTC), germination in vitro, and the peroxidase reaction. Only the peroxidase test was successful, and thus was used to detect pollen viability, and the other methods (FDA, TTC, and germination in vitro) were not used further. Pollen grains from freshly dehisced anthers were collected at 05:00–06:00, 06:00–7:00 and 07:00–08:00, respectively, on a sunny day in July (sunrise occurred at about 05:00 at the study site in July). All plants were growing in the field. The soultion for determination of pollen viability comprised two reagents, i.e. reagent I (1:1:1 0.5% benzidine: 0.5% α-naphthol: 0.25% sodium carbonate) and reagent II (0.3% hydrogen peroxide solution). The pollen grains were placed onto a glass slide that bore a culture medium comprising one drop of reagent I and one drop of reagent II, and then incubated at 30°C for 30 min. The pollen grains turned red if there were viable. The number of viable pollen grains in 10 optical fields was counted under an Olympus BX41 microscope and at least 50 pollen grains were found in each field. The pollen grains were collected from 10 flowers and five plants (two flowers per plant) and mixed together. Each cross was repeated three times, and approximately 2000 pollen grains were tested in each experiment.

### Artificial pollination

Lotus plants are insect-pollinated and the pistil matures one or two days before stamen maturation. In order to prevent insect pollination in the present study, we bagged the flowers of the female parents before the pistil matured. The optimal time for pollination is 05:00 to 08:00 on a sunny day. The pollen grains from 20 bagged male flowers from five plants (four flowers per plant) were collected between 05:00 and 06:00, and used for artificial pollination once the female flowers opened and their stigma surface was coated with bright yellow mucus. We used 150–200 flowers from about 50 plants as the pollen receptor for each cross. The immature stamens of the pollen-receptor flower were first removed, and then the stigmas were pollinated with the freshly collected pollen grains. After artificial pollination, the pollinated flowers were bagged again.

### Pollen behavior on the stigma after pollination

Pollen germination on the stigma in vivo was investigated as described by Sun et al. [13] with minor modifications. Twenty pistils per cross were fixed in FAA solution (5:5:90 formalin: acetic acid: 70% ethanol) at 0.5, 1, 2, 4, 6, 8, 10 and 12 h after pollination, respectively, then stored at 4°C until use. Each flower usually contained 20–35 pistils from which we randomly collected 20 pistils from three flowers at each time-point per cross. Pistils were collected from a total of 24 flowers per cross. The ovaries were removed and the stigmas softened overnight in 1 mol l^-1^ NaOH, rinsed in water and mounted on a microscope slide with a drop of 0.1% aniline blue (0.1 mol l^-1^ K_3_PO_4_ supplemented with 18% glycerol), and then observed under a fluorescence microscope (Zeiss Axioskop 40) with a BP 395-440 excitation filter BP 395-440, FT 460 chromatic beam splitter, and LP 470 barrier filter. Digital images were captured with an Axiocam MRC camera [11]. In addition, some pistils were fixed in 2.5% glutaraldehyde (0.1 M phosphate buffer, pH 7.2), dehydrated in an ethanol series (40, 70, 90 and 100%, for 15 min at each concentration), subjected to critical point drying and coated with gold for scanning electron microscopy (Hitachi S-3000 N), followed by digital processing of the images [14].

### Examination of embryo development and seed set

About 80 ovaries (or seeds) were collected from 10 flowers on five plants (two flowers per plant) for each pollination treatment at both 1 and 2 days after pollination and immediately immersed in FAA until use for examination of embryo development. The ovules were dissected from the ovaries and dehydrated through an alcohol series (70, 85, 95 and 100%, for 5 min at each concentration), then infiltrated with xylene, and embedded in paraffin wax [13]. Sections were cut to a thickness of 8-10 μm, stained in Heidenhain's haematoxylin, then observed and photographed under an Olympus BX41 microscope. In addition, we collected about 80 ovaries at 4, 6, 8 and 11 days after pollination for determination of the ratio of plump seeds to shriveled seeds over time. These samples were observed and photographed under a stereo microscope equipped with a digital cameral.

Among the pollinated flowers, we chose 30 flowers from 10 plants to determine seed set in each cross at 1 month after pollination. Seed set was calculated using the formula: seed set = (the number of plump seeds/the total number of pollinated stigmas) × 100% [13]. The crosses were performed in July and August, 2009, during which the average temperature was approximately 28°C (range 26–38°C). In addition, in order to investigate the percentage of normal pistils just before pollination, the seed set in emasculated flowers of female parents were determined under open pollination conditions. We also performed self-pollinations to determine self-compatibility of each cultivar. About 30 flowers from 10 plants per cultivar were bagged one day before the flowers opened, and the bags were immediately removed after the flowers withered. Furthermore, we emasculated 30 flowers from 10 plants, and these flowers were later pollinated with fresh pollen from other plants of the same cultivar.

### Statistical analysis

The data were subjected to a one-way analysis of variance using SPSS 16.0 (SPSS Inc, Chicago, IL, USA). The means were compared using the Bonferroni *t*-test with α = 0.05 (the type I experimentwise error rate). The data are presented as the mean value ± standard deviation.

## Results

### Pollen viability

The time of pollen grain collection had a significant effect on pollen viability (Table 1). The viability of pollen grains collected between 05:00 and 06:00 was 20.2 ± 1.1% for A, 16.2 ± 1.2% for G, 11.9 ± 2.1% for M, and 15.5 ± 1.6% for Q, respectively, significantly higher than for pollen collected between 06:00 and 08:00. Therefore, we collected pollen grains between 05:00 and 06:00 for the pollination experiments.

**Table 1:** Pollen viability of four water lotus cultivars determined with the peroxidase method at different collection times

**Collection time**	**Pollen viability (%)**			
	**A**	**G**	**M**	**Q**
05:00–06:00	20.2 ± 1.1a	16.2 ± 1.2a	11.9 ± 2.1a	15.5 ±1.6a
06:00–07:00	15.9 ± 0.5b	11.4 ± 0.8b	6.7 ± 0.9b	9.1 ± 1.0b
07:00–08:00	10.3 ± 0.9c	7.3 ± 0.6c	4.1 ± 0.3c	4.8 ± 0.6c

Values (mean ± standard deviation) followed by the same letter are not significantly different at α = 0.05 with the Bonferroni *t*-test. The pollen grains were collected from 10 flowers on five plants (two flowers per plant) and then mixed together for each cultivar. Approximately 2000 pollen grains were counted for each estimate. A, G, M and Q represent the lotus cultivars, ‘Aijiangnan’, ‘Guili’, ‘Molingqiuse’ and ‘Qinhuaiyanzhi’, respectively.

### Stigma receptivity

In the four crosses, the number of germinated pollen grains on each stigma increased during the 4 h after pollination; the highest number was attained 4 h after pollination, and thereafter gradually decreased. For example, 60.6, 25.8, 10.0 and 4.8 germinated grains per stigma were recorded 4 h after pollination in GA, AG, MQ and QM, respectively (Table 2). It was surprising that the number of pollen tubes decreased after 4 h, because even if no additional pollen grains germinated after this time, the number should remain constant and not decrease. A reasonable explanation is as follows. It usually takes 6–8 h for fertilization to be effected in *N. nucifera* flowers after pollen grains have landed on the stigma. In other words, pollen grains germinate quickly after they are deposited on the stigma. In general, most of the pollen grains germinated and their pollen tubes penetrated the stigmatic surface and grew towards the ovaries within 4 h. After fertilization of the ovules, the stigmas quickly become dry, which will render pollen tubes on the stigma fragile. Pollen tubes on stigmas collected at 8 and 12 h after pollination were increasingly fragile, thus they were more easily washed away during the processes of fixation in FAA, softening in 1 N NaOH, and washing in distilled water. As a consequence, the number of pollen tubes on the stigma decreased after 4 and did not remain constant in the present study.

**Table 2:** Number of germinated pollen grains on the stigma after pollination in four water lotus crosses

**Time after pollination (h)**	**Number of germinated pollen grains per stigma**			
	**GA**	**AG**	**MQ**	**QM**
0.5	15.0 ± 2.4d	8.4 ± 2.9c	3.0 ± 1.2c	1.0 ± 0.7d
2	31.2 ± 3.4b	16.6 ± 2.1b	7.8 ± 1.8b	3.2 ± 1.3bc
4	60.6 ± 4.6a	25.8 ± 3.2a	10.0 ± 3.2a	4.8 ± 2.4a
8	23.0 ± 2.6c	13.2 ± 2.7b	6.4 ± 1.8b	3.6 ± 1.1b
12	11.4 ± 2.3d	6.2 ± 1.9c	4.2 ± 1.6c	2.6 ± 1.1c

Values (mean ± standard deviation) followed by the same letter are not significantly different at α = 0.05 with the Bonferroni *t*-test. Twenty stigmas were used at each time in each cross, and these stigmas were randomly collected from three flowers (each flower usually had 20–35 stigmas) on three plants at each time-point. GA, ‘Guili’ (pollen receptor) × ‘Aijiangnan’ (pollen donor); AG, ‘Aijiangnan’ (pollen receptor) × ‘Guili’ (pollen donor); MQ, ‘Molingqiuse’ (pollen receptor) × ‘Qinhuaiyanzhi’ (pollen donor); QM, ‘Qinhuaiyanzhi’ (pollen receptor) × ‘Molingqiuse’ (pollen donor).

**Figure 1:**
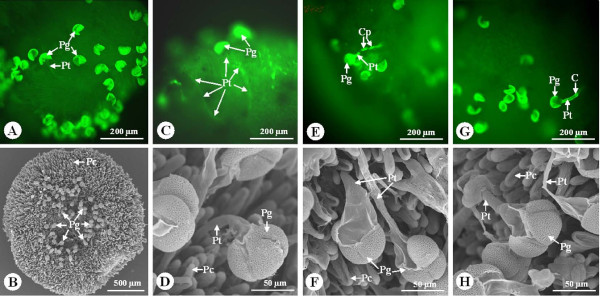
** Germination behavior of pollen grains on the stigma 4 h after pollination in*Nelumbo nucifera.*(A, B)** Many pollen grains germinated on the stigma in GA. ( **C, D)** pollen tubes penetrated the stigma and grew through the transmitting tissues of the style. **(E-H)** Some pollen grains germinated on the stigma in MQ, but callose was deposited in the pollen tubes or the pollen tubes were ruptured. Thus the pollen tubes failed to penetrate the stigma. C, Callose; Cp, callose plug; Pc, Papilla cell; Pg, pollen grain; Pt, pollen tube.

In the reciprocal crosses of G and A, most pollen tubes penetrated the stigma and grew through the transmitting tissues of the stigmas (Figure 1A-D). However, in the reciprocal crosses of M and Q, typical incongruity reactions were observed. Most of the pollen tubes exhibited abnormalities such as callose deposition and tube breakdown, which prevented some pollen tubes from penetrating the stigmatic surface and growing through the transmitting tissues of the style (Figure 1E-H).

### Embryo development

**Figure 2:**
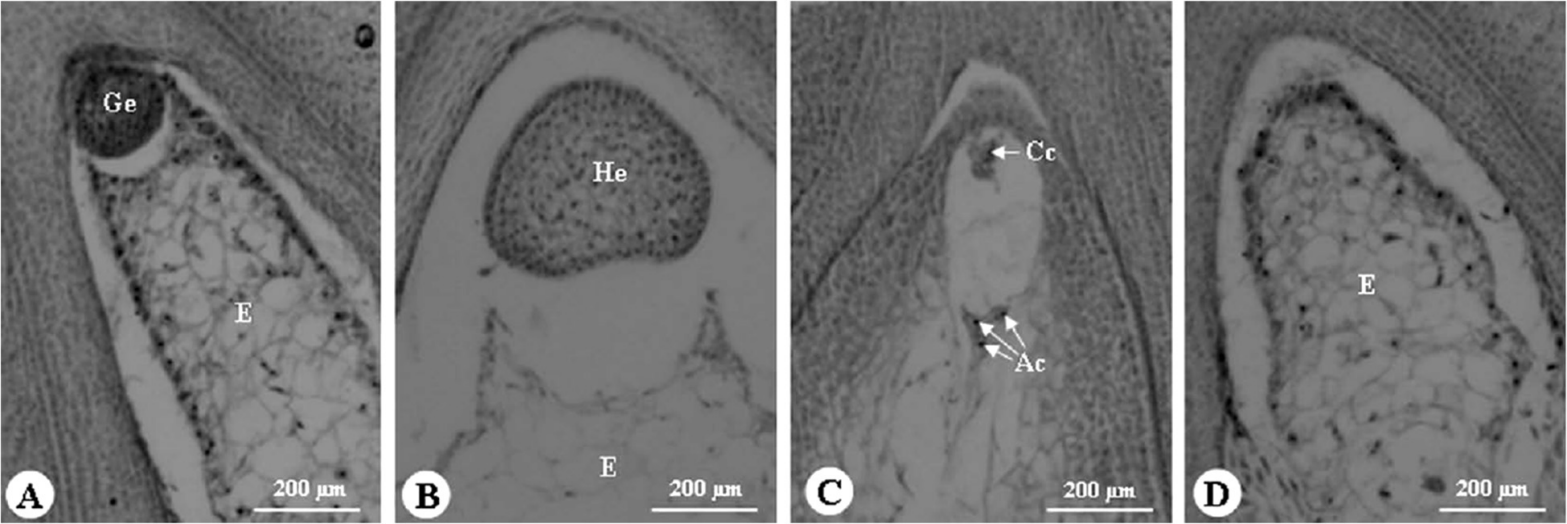
** Anatomical structure of the ovary of*Nelumbo nucifera*after pollination.(A)** Globular embryo at 1 day after pollination in GA. **(B)** Heart embryo 2 days after pollination in GA. **(C)** Failure of fertilization in MQ. **(D)** Embryo sac with only endosperm in MQ. Ac, antipodal cell; Cc, central cell; E, endosperm; He, heart embryo; Ge, globular embryo.

**Figure 3:**
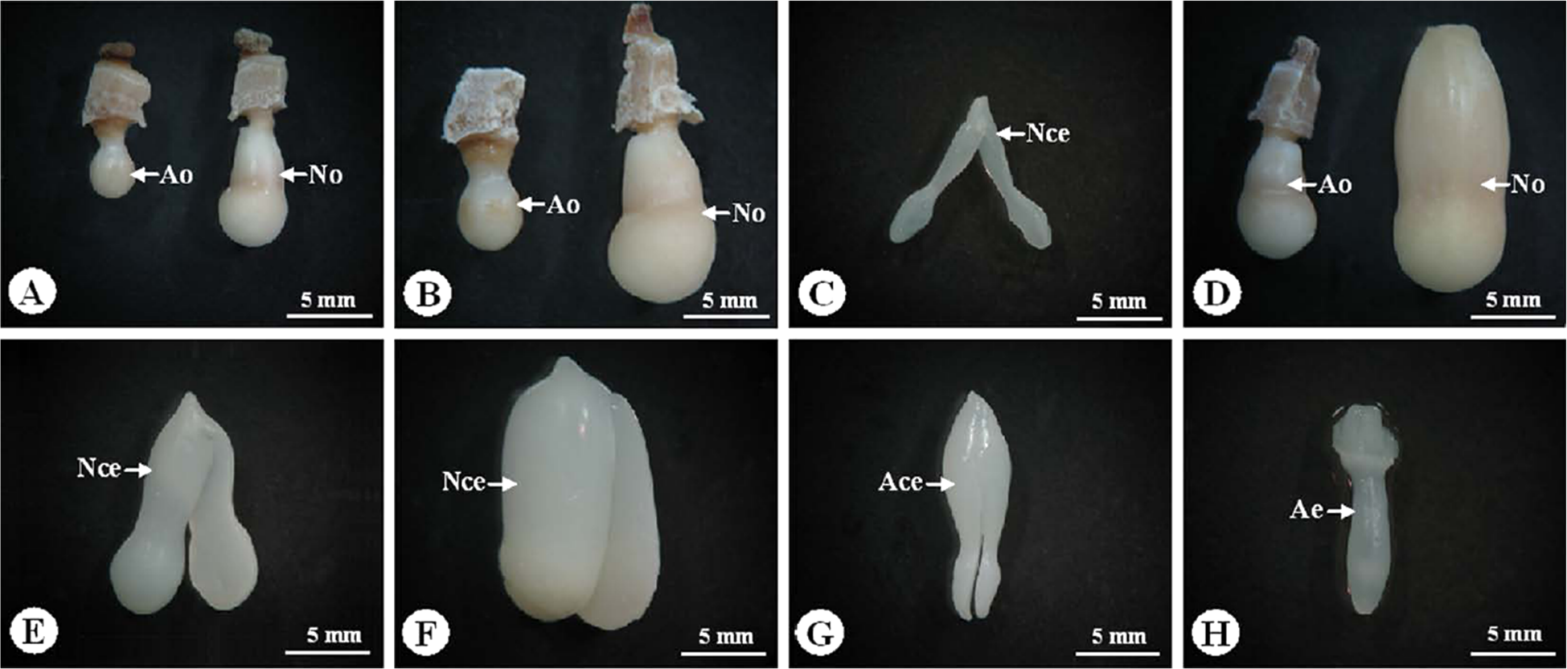
** Ovule and embryo morphology in*Nelumbo nucifera*after pollination.(A)** Normal and abnormal ovules 4 days after pollination. **(B)** Normal and abnormal ovules 6 days after pollination. **(C)** Normal cotyledon embryo 6 days after pollination. **(D)** Normal and abnormal ovules 8 days after pollination. **(E)** Normal cotyledon embryo 8 days after pollination. **(F)** Normal cotyledon embryo 11 days after pollination. **(G, H)** Abnormal embryos 11 days after pollination. Ace, abnormal cotyledon embryo; Ae, abnormal embryo; Ao, abnormal ovule; Nce, normal cotyledon embryo; No, normal ovule.

The zygote divided rapidly and a globular embryo was observed 1 day after pollination (Figure 2A). By 2 days after pollination, the zygote has developed into the heart-shaped embryo stage (Figure 2B). The zygote usually reached the cotyledon stage after 4 days of rapid cell divisions. Some eggs were not fertilized after pollination and many embryos aborted during the different stages of embryo development in the four crosses (Table 3; Figure 2C, D; Figure 3). For example, the percentage of normal embryos at 1 day after pollination in GA, AG, MQ and QM was 58.8%, 26.8%, 9.6% and 7.6%, respectively; at 2 days after pollination, 44.4%, 20.5%, 5.2% and 4.1%, respectively; and at 11 days after pollination, 25.0%, 11.3%, 1.5% and 0%, respectively. In the reciprocal crosses of M and Q, the percentages of normal embryos at the different stages of embryo development were lower than those in the reciprocal crosses of G and A (Table 3).

**Table 3:** Number of normal and abnormal embryos at different days after pollination in four water lotus crosses

**Crosses**	**Days after pollination**	**Developmental stages of embryo**	**Percentage of normal embryo (%)**
GA	1	Globular embryo	58.8 ± 6.3a
	2	Heart embryo	44.4 ± 5.6b
	4	Cotyledon embryo	30.8 ± 4.1c
	11		25.0 ± 3.8d
AG	1	Globular embryo	26.8 ± 3.3a
	2	Heart embryo	20.5 ± 2.8b
	4	Cotyledon embryo	15.2 ± 2.1c
	11		11.3 ± 2.4d
MQ	1	Globular embryo	9.6 ± 1.9a
	2	Heart embryo	5.2 ± 1.2b
	4	Cotyledon embryo	4.7 ± 1.4b
	11		1.5 ± 0.3c
QM	1	Globular embryo	7.6 ± 1.8a
	2	Heart embryo	4.1 ± 1.1b
	4	Cotyledon embryo	3.1 ± 0.5b
	11		0c

Values (mean ± standard deviation) followed by the same letter are not significantly different at α = 0.05 with the Bonferroni *t*-test. GA, ‘Guili’ (pollen receptor) × ‘Aijiangnan’ (pollen donor), AG, ‘Aijiangnan’ (pollen receptor) × ‘Guili’ (pollen donor); MQ, ‘Molingqiuse’ (pollen receptor) × ‘Qinhuaiyanzhi’ (pollen donor); QM, ‘Qinhuaiyanzhi’ (pollen receptor) × ‘Molingqiuse’ (pollen donor).

### Seed set

Seed set varied greatly among the four crosses. It was 23.33% in GA and 9.24% in AG. The percentage in MQ was 3.11%, and only one seed was obtained in QM (0.2% of 427 ovules pollinated). Seed set of most female parents under open-pollination conditions was higher than that in the corresponding cross. Our observations indicated that bees, flies and beetles may be effective pollinators because they were the most frequent floral visitors. Seed set for G, A, M, and Q under open-pollination conditions was 26.5%, 34.9%, 11.5%, and 8.4%, respectively (Table 4), which indicated that a high proportion of pistils of G and A were normal just before pollination, and that seed set under open-pollination conditions was higher than that under artificial pollination. The higher levels of fruit set under open-pollination conditions may be attributable to deposition of a mixture of pollen grains from the 30 cultivars growing at the study site, because pollen mixtures often increase fertilization success by increasing recognition between pollen grains and stigmas. No seeds developed after self-pollination of each cultivar, which indicated that the four cultivars were self-incompatible.

**Table 4:** Seed set after artificial pollination in four water lotus crosses and in emasculated flowers of each cultivar under open-pollination conditions

**Seed set after artificial pollination (%)**	**Seed set under open-pollination condition (%)**
23.3 ± 3.5 (GA)	26.5 ± 4.8 (G)
9.2 ± 1.7 (AG)	34.9 ± 6.1 (A)
3.1 ± 0.6 (MQ)	11.5 ± 2.7 (M)
0.2 ± 0.16 (QM)	8.4 ±2.1 (Q)

The data are presented as the mean value ± standard deviation. GA, ‘Guili’ (pollen receptor) × ‘Aijiangnan’ (pollen donor); AG, ‘Aijiangnan’ (pollen receptor) × ‘Guili’ (pollen donor); MQ, ‘Molingqiuse’ (pollen receptor) × ‘Qinhuaiyanzhi’ (pollen donor); QM, ‘Qinhuaiyanzhi’ (pollen receptor) × ‘Molingqiuse’ (pollen donor). G, A, M and Q represent the four lotus cultivars ‘Guili’, ‘Aijiangnan’, ‘Molingqiuse’ and ‘Qinhuaiyanzhi’, respectively.

## Discussion

Many factors affect seed set of angiosperms. Pollen viability is often considered to be an important factor that influences seed or fruit production [13,15-18]. When pollen grains with low viability are placed on a stigma, the probability of pollination failure usually increases, resulting in reduced seed production [17,19,20]. For example, in *Bambusa vulgaris*, low pollen viability is one of main factors that causes low seed set [21]. In an investigation into the relationship between soybean production and pollen sterility, it was noticed that soybean production significantly decreased when more than 60% of the pollen grains were sterile [19]. In the present study, the time of lotus pollen grain collection significantly influenced pollen viability. Pollen grains collected between 05:00 and 06:00 contained the highest percentage of viable grains, and consequently these grains were used for the artificial pollinations. The viability of pollen of the four cultivars collected between 05:00 and 06:00 ranged from approximately 10% to 20%, which might be somewhat lower than the actual values. A possible explanation is that the pollen viability of *N. nucifera* quickly decreases after anther dehiscence [3], and it usually took about 2 h to transport the samples from the study site to our laboratory. It is possible that a portion of the pollen grains lost viability during transport. Even if the actual pollen viability of the four cultivars was slightly higher than 20%, seed set may be related to low parental pollen viability.

Pollination is the first step in the sexual reproduction process for most of higher plants, and the interaction between pollen grains and the pistil after pollination is an important factor that influences seed output [22-27]. Pollination failure is widespread in plants, and is a common cause of low seed production. It is often characterized by inhibition of pollen germination and pollen tube growth, and abnormal growth of pollen tubes [13,17,26,27]. For example, low stigma receptivity is considered to be one cause of low seed set in some sorghum crosses [28]. More recently, in a study of the factors that affect seed set in several chrysanthemum crosses, we found that a low number of germinated pollen grains, and the abnormal growth of most pollen tubes, were the main causes of the failure of the cross between *D. grandiflorum* ‘Yuhuaxingchen’ and *D. nankingense*[13]. The present results show very slight differences in pollen viability among the four cultivars, but significant differences in the number of germinated pollen grains on the stigma at 4 h after pollination among the four crosses. In addition, the number of germinated pollen grains on the stigma in each cross was positively correlated with the percentages of normal globular embryos at 1 day after pollination and seed set. Moreover, in the reciprocal crosses of M and Q, large amounts of callose were deposited in some pollen tubes, and some pollen tubes were ruptured, which resulted in failure of the pollen tubes to penetrate the stigma. Therefore it is suggested that low seed set in the reciprocal crosses of M and Q may be largely attributable to low numbers of germinated pollen grains and subsequent abnormal growth of their pollen tubes on the stigma. Because pollen–pistil interactions are complex, usually including cell–cell recognition and cellular signaling processes [19,26], further investigation into the causes of pollen germination inhibition and pollen tube growth are necessary in order to increase stigmatic receptivity in water lotus crosses.

In addition to pollen viability and pollen–pistil interactions, seed set is closely related to embryo development. Reduced seed production is often caused by degeneration or abortion of a large number of embryos during their development [12,29-32]. For example, Ndoutoumou et al. [12] found that low seed set in reciprocal crosses between *Phaseolus vulgaris* and *P. coccineus* was largely because of embryo abortion after fertilization. Similarly, abortion of immature embryos is considered to be the main factor restricting the transfer of desired traits into cultivated chickpea from wild *Cicer* relatives [31]. Recently, similar phenomena have been observed in wide crosses of chrysanthemum [13]. Likewise, embryo abortion is also a factor affecting seed set in water lotus crosses, although the proportion of aborted embryo is variable in these crosses. Compared with the three other crosses performed in the present study, GA had a relatively lower ratio of embryo abortion and a higher seed set (23.33%). If seed set of over 10% is regarded as a criterion for a successful water lotus cross, the three crosses, AG, MQ, and QM were all unsuccessful. We suggest that embryo abortion is an important factor leading to the failure of the AG, MQ, and QM crosses and influences the breeding efficiency of GA. The reasons for embryo abortion, however, are still unclear and require further investigation.

Seed set in GA was 23.3% but was only 9.2% in AG, which was similar to the seed set in the reciprocal crosses of M and Q. This difference in seed set in one reciprocal cross may be mainly attributable to differences in pistil receptivity. For example, the number of germinated pollen grains on the stigma at 4 h after pollination was 60.6 in GA and 25.8 in AG, which is a 2.35–fold discrepancy. The percentage of normal embryos at 1 d after pollination in GA was 58.8%, which was 2.21–fold higher than that in AG (26.6%), and seed set in GA was 2.53–fold higher than that in AG. The reasons for the differences in pistil receptivity in one reciprocal cross remain unclear.

## Conclusion

In this paper, we carefully studied the factors that govern fecundity in two lotus reciprocal crosses. Three findings in the present study are of note: i) low pollen viability impacts on seed set in water lotus crosses; ii) low stigma receptivity is a vital factor leading to low fecundity of water lotus crosses; and iii) embryo abortion is an additional factor leading to reduced fecundity of lotus crosses. However, the mechanisms involved in low stigma receptivity and embryo abortion remain unknown, and further studies are required to better understand these factors in water lotus, and indeed in other plants as well.

## Authors' contributions

NJT, WMF and FDC designed the experiments. NJT, WYL, and CQS performed the experiments and analyzed the data. NJT, YLW, WMF and FDC wrote and revised the manuscript. All authors read and approved the final manuscript.
